# The SARS-CoV-2 Virus and the Cholinergic System: Spike Protein Interaction with Human Nicotinic Acetylcholine Receptors and the Nicotinic Agonist Varenicline

**DOI:** 10.3390/ijms24065597

**Published:** 2023-03-15

**Authors:** Eric C. Carlson, Marian Macsai, Sonia Bertrand, Daniel Bertrand, Jeffrey Nau

**Affiliations:** 1 Oyster Point Pharma, Inc., Princeton, NJ 08540, USA; mmacsai@oysterpointrx.com (M.M.); jnau@oysterpointrx.com (J.N.); 2 HiQScreen Sàrl, 1222 Geneva, Switzerland; sonia.bertrand@hiqscreen.com (S.B.); daniel.bertrand@hiqscreen.com (D.B.)

**Keywords:** SARS-CoV-2, human nicotinic acetylcholine receptors, protein interaction

## Abstract

Severe acute respiratory syndrome coronavirus-2 (SARS-CoV-2) is responsible for the worldwide coronavirus disease 2019 (COVID-19) pandemic. Although the pathophysiology of SARS-CoV-2 infection is still being elucidated, the nicotinic cholinergic system may play a role. To evaluate the interaction of the SARS-CoV-2 virus with human nicotinic acetylcholine receptors (nAChRs), we assessed the in vitro interaction of the spike protein of the SARS-CoV-2 virus with various subunits of nAChRs. Electrophysiology recordings were conducted at α4β2, α3β4, α3α5β4, α4α6β2, and α7 neuronal nAChRs expressed in *Xenopus* oocytes. In cells expressing the α4β2 or α4α6β2 nAChRs, exposure to the 1 µg/mL Spike-RBD protein caused a marked reduction of the current amplitude; effects at the α3α5β4 receptor were equivocal and effects at the α3β4 and α7 receptors were absent. Overall, the spike protein of the SARS-CoV-2 virus can interact with select nAChRs, namely the α4β2 and/or α4α6β2 subtypes, likely at an allosteric binding site. The nAChR agonist varenicline has the potential to interact with Spike-RBD and form a complex that may interfere with spike function, although this effect appears to have been lost with the omicron mutation. These results help understand nAChR’s involvement with acute and long-term sequelae associated with COVID-19, especially within the central nervous system.

## 1. Introduction

The coronavirus disease 2019 (COVID-19) pandemic was caused by severe acute respiratory syndrome coronavirus-2 (SARS-CoV-2), which has continuously evolved and mutated into a range of variants with differing transmission and pathogenesis characteristics [1]. One approach to help understand acute and long-term sequelae associated with SARS-CoV-2 infection, particularly the neurological consequences [2], is to investigate the complex binding interactions of the SARS-CoV-2 virus with various receptors in the human body.

The SARS-CoV-2 virus envelope contains a spike (S) protein of 1273 amino acids, which is indispensable for attachment to its host cell. This S protein consists of S1 and S2 subunits that are cleaved by target cell proteases during viral infection [3]; upon cleavage, the S2 protein mediates fusion to and entry into the target cell [4]. Although much of the focus of the SARS-CoV-2 interaction with the human body has been on the angiotensin-converting enzyme-2 (ACE2) receptor, coronaviruses have been shown to interact with other human cellular receptors [5]. The nicotinic cholinergic system has been implicated in the pathophysiology of SARS-CoV-2 [6,7,8], supported in part by observations in meta-analyses of the unexpectedly low prevalence of smokers among hospitalized patients with SARS-CoV-2 [9,10,11]. In addition, molecular simulations indicate that there may be an interaction between the Y674-R685 region of the S protein with select nicotinic acetylcholine (ACh) receptors (nAChRs) [12]. nAChRs are ligand-gated ion channels that can be found distributed widely throughout the human body, including at the skeletal neuromuscular junction, in the brain, on peripheral neurons, in the gastrointestinal tract, and on organs such as the heart [13].

Varenicline is a full nAChR agonist at the α7 receptor and a partial agonist at the α4β2, α4α6β2, α3β4, and α3α5β4 receptors. Varenicline was recently shown to have in vitro antiviral activity against wildtype, alpha, and beta variants of the SARS-CoV-2 virus in multiple cell lines (Calu-3 and Caco-2 cells), as well as in vivo antiviral activity in a rhesus macaque model of SARS-CoV-2 nasal infection [14]. Other studies have suggested that the SARS-CoV-2 virus may interact with the α4β2, α3β4, and α7 nAChR subtypes [7,15,16,17]. Therefore, characterizing how the SARS-CoV-2 S protein and S1 and S2 subunits interact with human nAChRs would help elucidate whether the nicotinic ACh system has a role in the pathophysiology of SARS-CoV-2 infection. In particular, interaction with select nAChR subtypes may provide insight into the specific mechanisms of action of SARS-CoV-2, as well as the long-term sequelae of this infection.

## 2. Results

### 2.1. Spike-RBD Interacts with the Human α4β2 and α4α6β2 nAChRs and Possibly the α3α5β4 nAChR, but Not the α3β4 and α7 nAChRs

**Figure 1:**
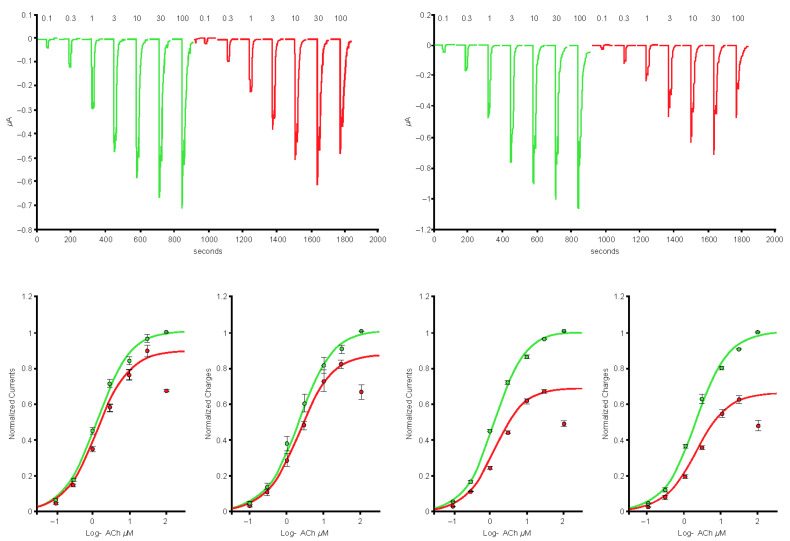
Effects of the spike protein containing the receptor-binding domain (Spike-RBD) protein at the concentration-response curve to acetylcholine (ACh) of the α4β2 nicotinic acetylcholine receptor. Typical currents were recorded in the same cell before (green traces) and during short (left-hand side) and long (right-hand side) exposure to 1 μg/mL of the Spike-RBD protein (red traces). Currents were elicited by brief exposure to ACh test pulses with a series of concentrations. Currents were normalized to unity vs. the maximal value and average results obtained in seven cells were plotted vs. the logarithm (log) of the ACh concentration. The green curve is the best fit obtained with the Hill equation. Areas under the curve are plotted on the lower right-hand side.

**Figure 2:**
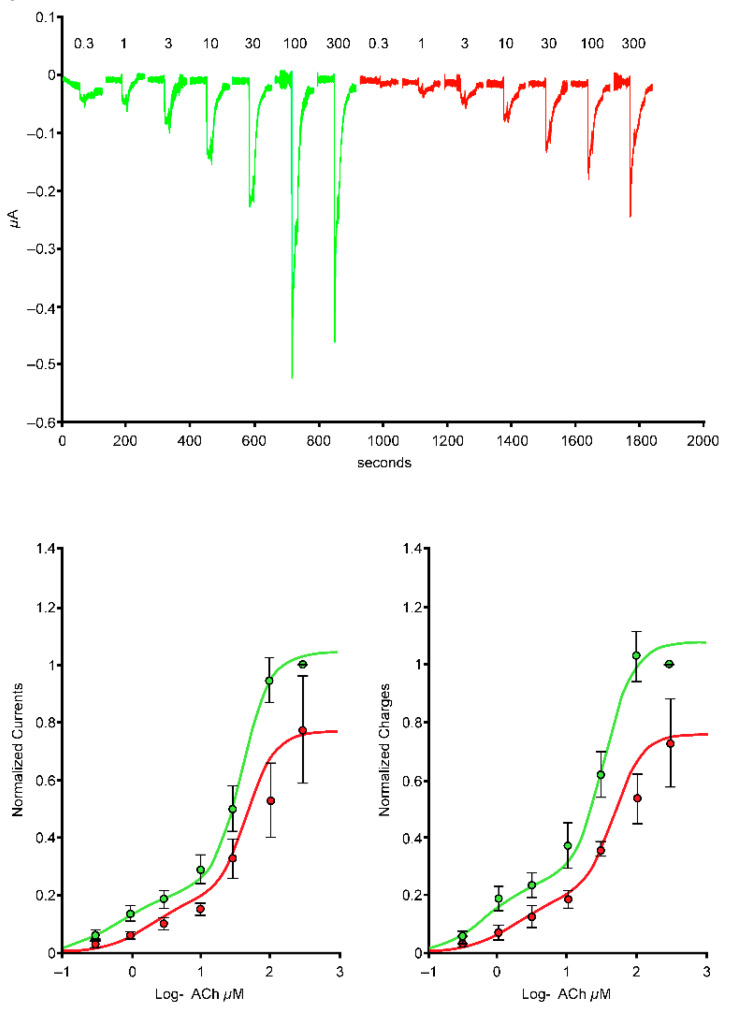
Effects of the spike protein containing the receptor-binding domain (Spike-RBD) protein at the concentration-response curve to acetylcholine (ACh) of the α4α6β2 nicotinic acetylcholine receptor complementary DNA. Typical currents were recorded in the same cell before (green traces) and during exposure to 1 μg/mL of the Spike-RBD protein (red traces). Currents were elicited by brief exposure to ACh test pulses with a series of concentrations. Currents were normalized to unity vs. the maximal value and average results obtained in five cells were plotted vs. the logarithm (log) of the ACh concentration. The green curve is the best fit obtained with the Hill equation. Areas under the curve are plotted on the lower right-hand side.

**Figure 3:**
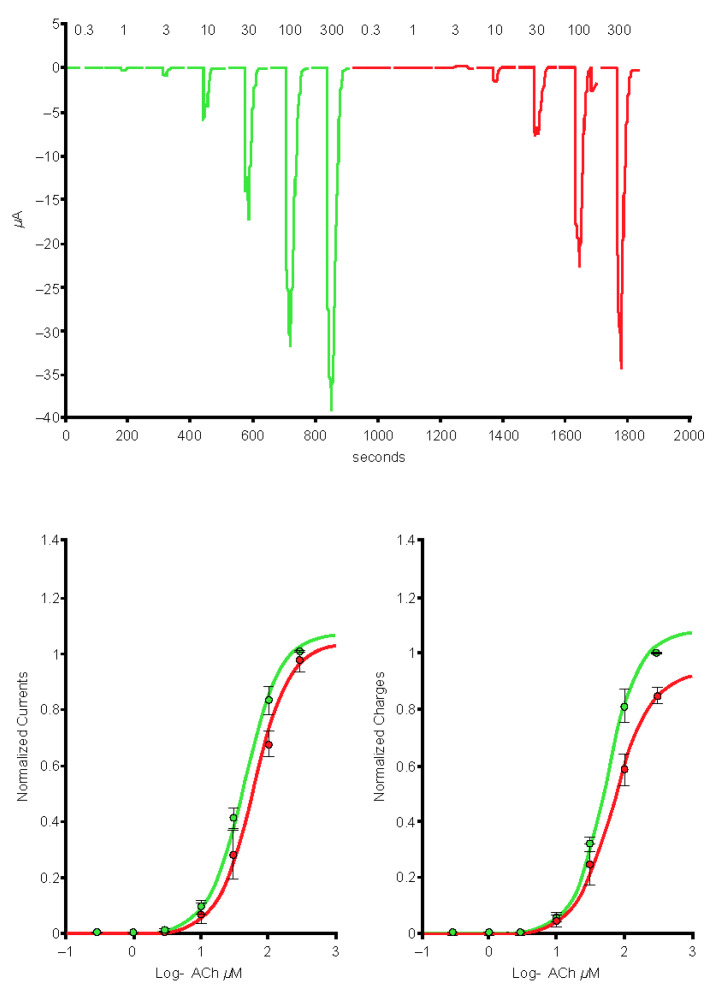
Effects of the spike protein containing the receptor-binding domain (Spike-RBD) protein at the concentration-response curve to acetylcholine (ACh) of the α3α5β4 nicotinic acetylcholine receptor. Typical currents were recorded in the same cell before (green traces) and during exposure to 1 μg/mL of the Spike-RBD protein (red traces). Currents were elicited by brief exposure to ACh test pulses with a series of concentrations. Currents were normalized to unity vs. the maximal value and average results obtained in four cells were plotted vs. the logarithm (log) of the ACh concentration. The green curve is the best fit obtained with the Hill equation. Areas under the curve are plotted on the lower right-hand side.

In cells expressing the α4β2 nAChRs (*n* = 7), exposure to the 1-µg/mL S protein containing the receptor-binding domain (Spike-RBD) protein caused a marked reduction of the current amplitude (Figure 1, left-hand side), and reduction was larger when cells were exposed over a longer period (Figure 1, right-hand side). In cells expressing the α4α6β2 nAChRs (*n* = 5), exposure to the 1-µg/mL Spike-RBD protein caused a marked reduction of the current amplitude (Figure 2). The current evoked at saturating ACh concentration was reduced by >20%. Determination of the α3α5β4 nAChR concentration-response curves in the absence or presence of 1 µg/mL of the Spike-RBD protein (*n* = 4) revealed a slight modification of the ACh response amplitude (Figure 3). Determination of the α7 (*n* = 8) and α3β4 (*n* = 5) nAChR concentration-response curves in the absence or presence of 1 µg/mL of the Spike-RBD protein revealed no significant modification of the ACh response amplitude or changes that occurred over the time course of the assessment. 

### 2.2. The S2 Subunit of the S Protein Interacts with the Human α4β2 nAChR, but Not the α4α6β2, α3β4, and α3α5β4 nAChRs

**Figure 4:**
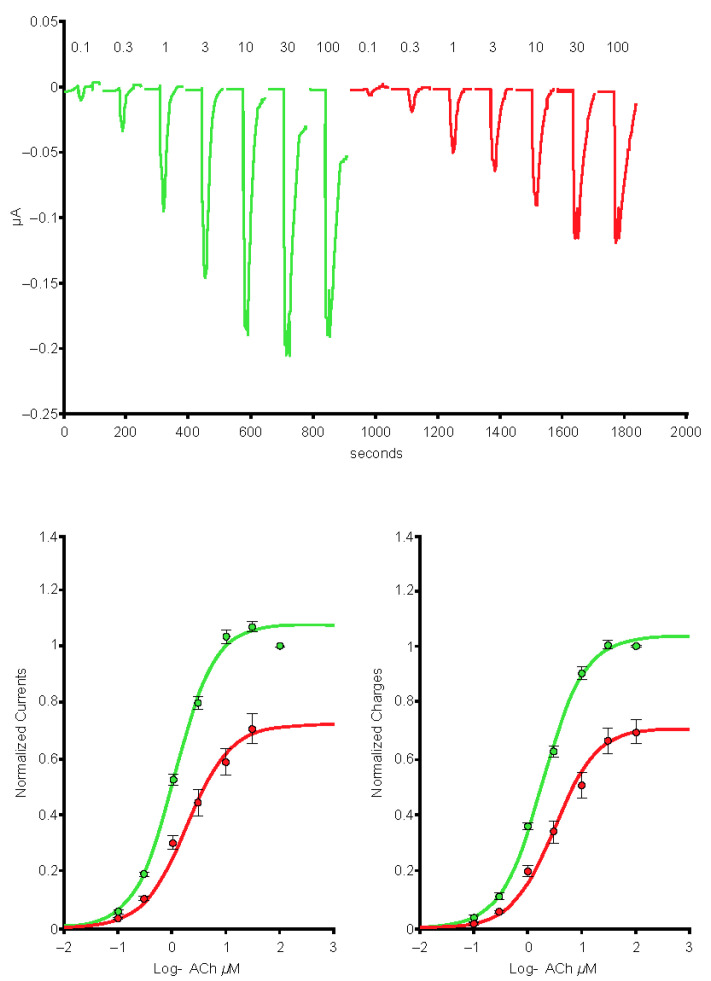
Effects of the SARS-CoV-2 spike protein S2 subunit at the human α4β2 nicotinic acetylcholine receptor. Typical currents were recorded in the same cell before (green traces) and during exposure to 1 μg/mL of the Spike protein S2 subunit (red traces). Currents were elicited by brief exposure to acetylcholine (ACh) test pulses with a series of concentrations. Currents were normalized to unity vs. the maximal value and average results obtained in nine cells were plotted vs. the logarithm (log) of the ACh concentration. The green curve is the best fit obtained with the Hill equation. Areas under the curve are plotted on the lower right-hand side.

In cells expressing the α4β2 nAChRs (*n* = 9), exposure to 1 µg/mL of the S2 protein caused a marked reduction of the current amplitude, consistent with the results seen with the Spike-RBD protein (Figure 4). Determination of the α3β4 (*n* = 3) and α3α5β4 (*n* = 4) nAChR concentration-response curves in the absence or presence of 1 µg/mL of the S2 protein revealed no significant modification of the ACh response amplitude or changes that occurred over the time course of the assessment. 

### 2.3. The S1 Subunit of the S Protein Interacts with the Human α4β2 nAChRs at an Allosteric Binding Site That Inhibits Receptor Activity

**Figure 5:**
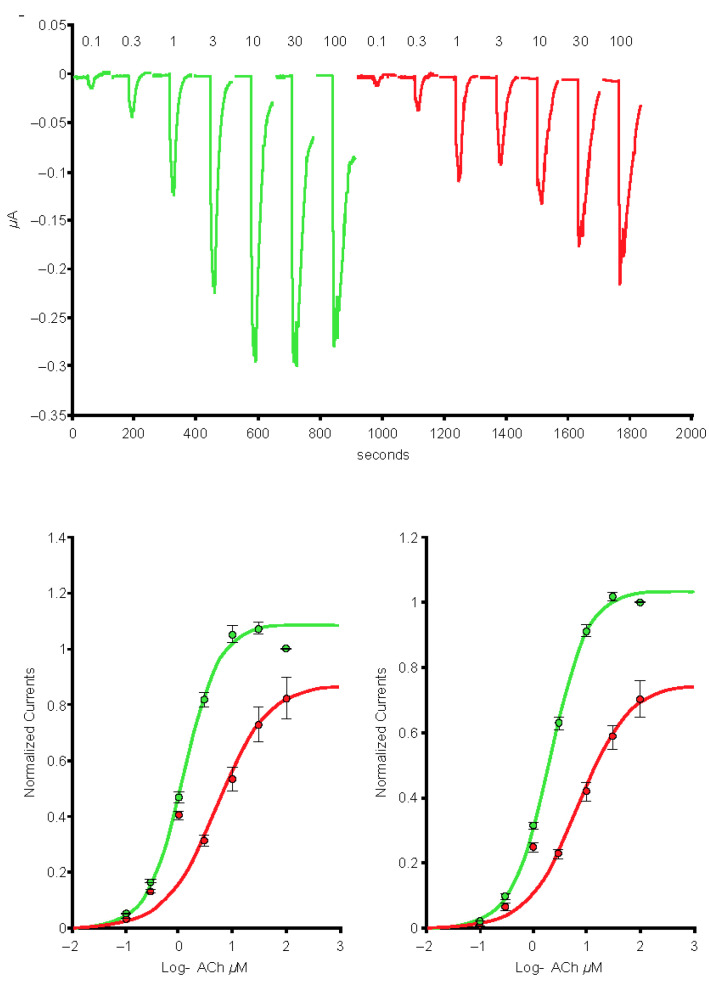
Effects of SARS-CoV-2 Spike protein S1 subunit at the human α4β2 nicotinic acetylcholine receptor. Typical currents were recorded in the same cell before (green traces) and during exposure to 1 μg/mL of the Spike protein S1 subunit (red traces). Currents were elicited by brief exposure to acetylcholine (ACh) test pulses with a series of concentrations. Currents were normalized to unity vs. the maximal value and average results obtained in six cells were plotted vs. the logarithm (log) of the ACh concentration. The green curve is the best fit obtained with the Hill equation. Areas under the curve are plotted on the lower right-hand side.

In cells expressing the α4β2 nAChRs (*n* = 6), exposure to 1 μg/mL of the S1 protein caused a marked reduction of the current amplitude (Figure 5), consistent with the results seen with the Spike-RBD and S2 proteins.

### 2.4. The SARS-CoV-2 B.1.1.529 Spike-RBD Protein Interacts with the Human α4β2 nAChR at an Allosteric Binding Site That Potentiates Receptor Activity

**Figure 6:**
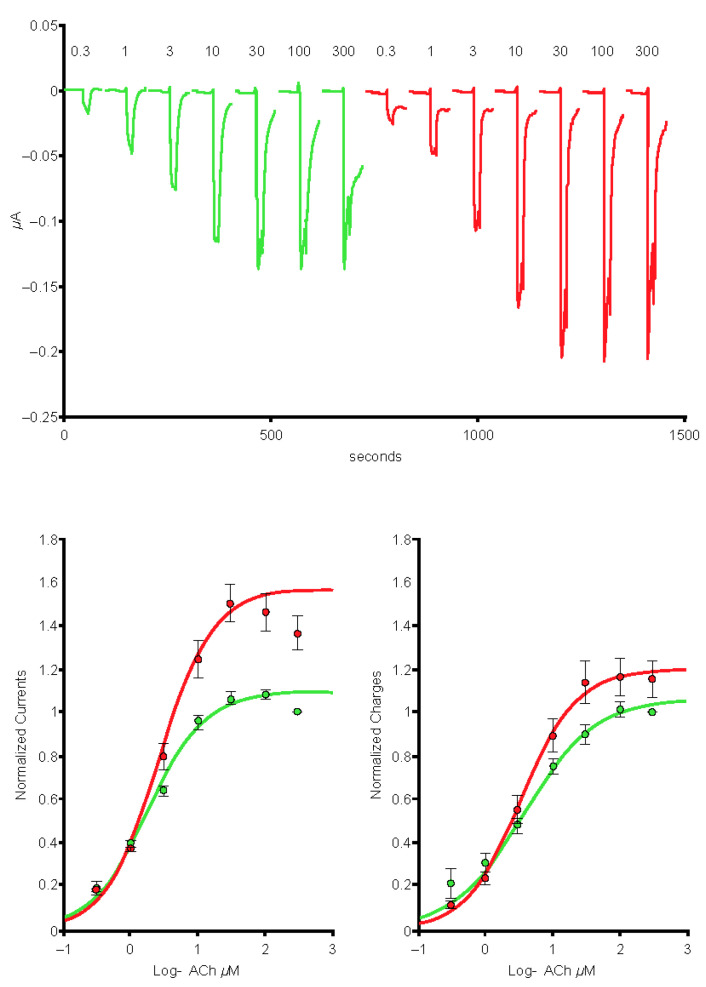
Effects of the severe acute respiratory syndrome coronavirus-2 (SARS-CoV-2) B.1.1.529 spike protein containing the receptor-binding domain (Spike-RBD) protein at the human α4β2 nicotinic acetylcholine receptor. Typical currents were recorded in the same cell before (green traces) and during exposure to 10 μg/mL of the SARS-CoV-2 B.1.1.529 Spike-RBD protein (red traces). Currents were elicited by brief exposure to acetylcholine (ACh) test pulses with a series of concentrations. Currents were normalized to unity vs. the maximal value and average results obtained in five cells were plotted vs. the logarithm (log) of the ACh concentration. The green curve is the best fit obtained with the Hill equation. Areas under the curve are plotted on the lower right-hand side.

In cells expressing the α4β2 nAChR (*n* = 5), exposure to 10 µg/mL of the SARS-CoV-2 B.1.1.529 Spike-RBD protein caused a marked potentiation of the current amplitude (Figure 6).

### 2.5. Preincubation of Varenicline with Spike-RBD Indicates That Varenicline Inhibition of the Human α4β2 nAChR Is Modified by Spike-RBD

**Figure 7:**
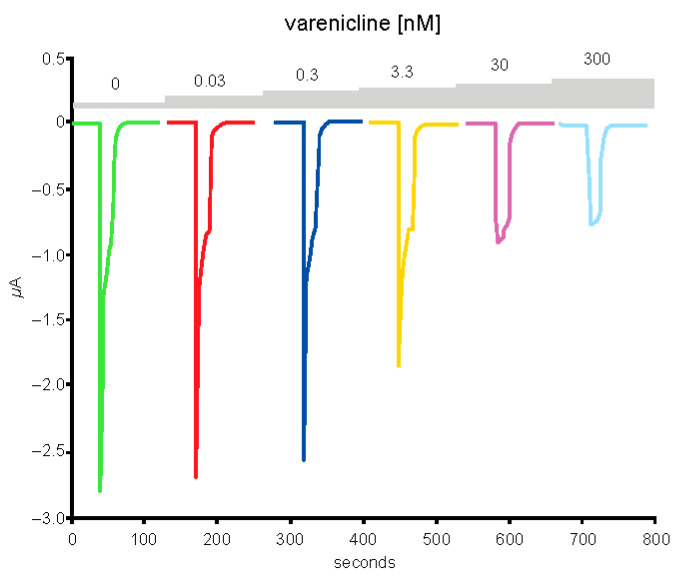
Exposure to low, but sustained, applications of varenicline desensitize the human α4β2 nicotinic acetylcholine receptor. Typical currents evoked by brief acetylcholine test pulses (50 μM) were recorded in a cell expressing the complementary DNA encoding for the human α4β2 receptors were recorded first in control conditions (green trace) and then during exposure to a series of varenicline concentrations ranging from 0.03 to 300 nM.

**Figure 8:**
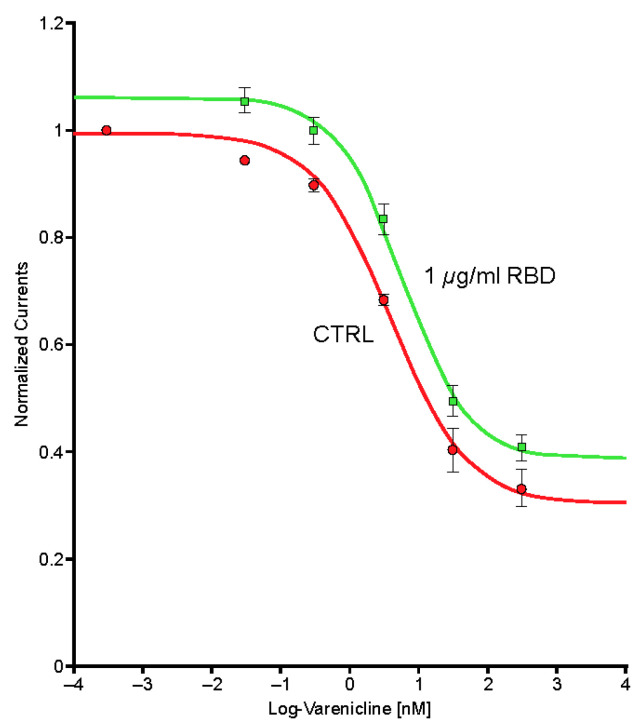
Peak inward currents evoked by 50-μM acetylcholine were recorded in a series of cells for control (*n* = 7; red) and during exposure to 1-μg/mL spike protein containing the receptor-binding domain (Spike-RBD) (*n* = 8; green) and normalized to unity vs. the response recorded in control. Continuous lines are the best fits obtained with the Hill equation. CTRL, control; log, logarithm.

**Figure 9:**
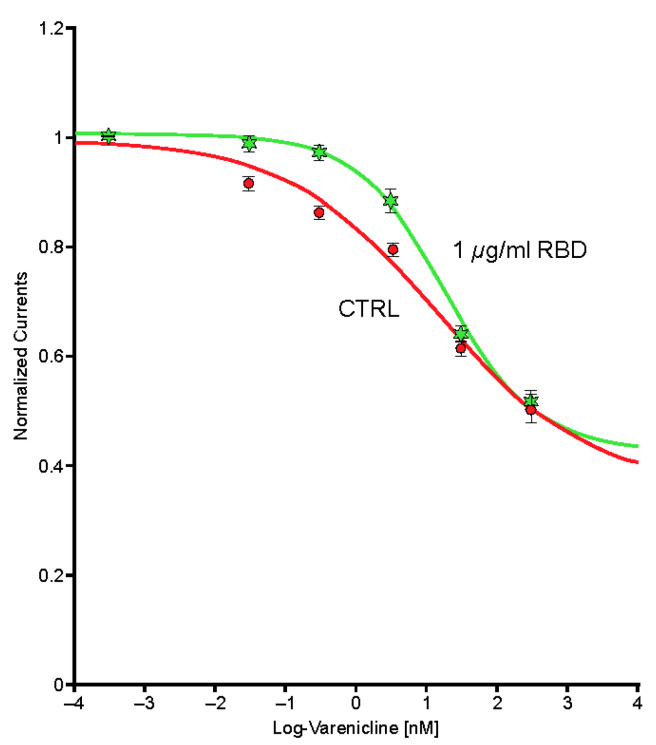
The area under the curve (charges) of the currents evoked by 50-μM acetylcholine recorded in a series of cells for control (*n* = 7; red) and during exposure to 1-μg/mL spike protein containing the receptor-binding domain (Spike-RBD) (*n* = 8; green) and normalized to unity vs. the response recorded in control. Continuous lines are the best fits obtained with the Hill equation. CTRL, control; log, logarithm.

Typical currents recorded in a cell (*n* = 1) expressing the human α4β2 nAChR exposed to a series of varenicline concentrations demonstrated a reduction in ACh-evoked current as a function of the varenicline concentration, indicating desensitization of the α4β2 receptor (Figure 7). Plots of the peak inward current as a function of the logarithm of the varenicline concentration recorded in a series of control cells (*n* = 7) and in a series of cells co-exposed to 1 µg/mL of the Spike-RBD protein (*n* = 8) are shown in Figure 8. The right shift caused by Spike-RBD protein exposure suggested a reduction of the efficiency of varenicline to desensitize the receptor, and it can be hypothesized that the interaction of the Spike-RBD protein with varenicline combines to yield a nonfunctional complex. Analysis of the area under the curve, which corresponds to the charges carried through the open receptors, constitutes an additional method to evaluate the possible interaction between varenicline and the Spike-RBD protein. A marked reduction in the area under the curve was observed for low concentrations of varenicline when cells were co-exposed to 1 µg/mL of the Spike-RBD protein (Figure 9), in agreement with the hypothesis of an interaction between varenicline and Spike-RBD.

### 2.6. The SARS-CoV-2 B.A.1 (Omicron) Spike-RBD Protein Interacts with the Human α4β2 nAChRs at an Allosteric Binding Site That Potentiates Receptor Activity

**Figure 10:**
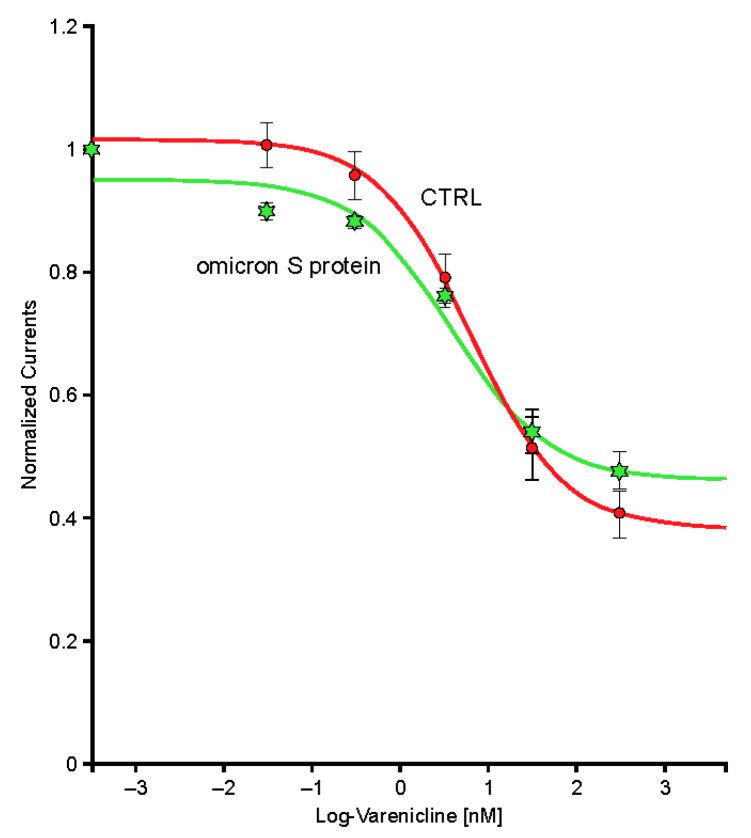
Peak inward currents evoked by 50-µM acetylcholine were recorded in a series of cells for control (*n* = 9; red) and during exposure to 10-µg/mL omicron S protein (*n* = 9; green) and normalized to unity vs. the response recorded in control. Continuous lines are the best fits obtained with the Hill equation. log, logarithm.

Exposure to the omicron S protein enhanced the response to ACh but did not cause significant modification of the desensitization caused by sustained exposure to low concentrations of an agonist such as varenicline (Figure 10).

## 3. Discussion

To the best of our knowledge, these in vitro studies are the first to attempt to elucidate the potential interaction of the S protein of the SARS-CoV-2 virus with human neuronal nAChRs using a functional electrophysiology model. In summary, we found that the S protein of SARS-CoV-2 interacted with some (α4β2 or α4α6β2 subtypes), but not all (the tested α3 or α7 subtypes), nAChRs. The lack of interaction at the homomeric α7 subtype is consistent with other research findings in the literature [17]. However, desensitization of the receptor was lost with the omicron mutation of the S protein (potentiation of the receptor was observed). In addition, this study found that an interaction exists between the SARS-CoV-2 S protein and the nicotinic agonist varenicline; these may form a nonfunctional complex that restricts binding at α4-containing nAChRs.

Although many studies have focused on the ability of SARS-CoV-2 to bind to ACE2 receptors and enter the cells to begin replication, the virus may have the ability to interact with other membrane proteins. Here we illustrate, using a functional electrophysiology model, that the S protein of the SARS-CoV-2 virus inhibits certain nAChR subtypes. This is consistent with computational binding studies that have suggested the Y674-R685 region of the S protein adopts particular conformation when binding to the α4β2 and α7 nAChR subtypes, although in our in vitro study, binding to the heteromeric α7 nAChR subtype was not detected [7]. Interaction with the α4 subunit-containing receptors throughout the body may explain some of the effects of acute viral infection or long-term symptoms, referred to as “long COVID” [18]. For example, one of the most common symptoms reported during and after acute viral infection is loss of taste and/or smell, and nAChRs are located on the olfactory bulb in the nasal cavity and taste receptor cells in the oral cavity [19,20]. Another example is that symptoms of brain fog, headache, dizziness, and weakness are not dissimilar to those experienced during exposure to large doses of nicotine from harvesting tobacco leaves [21]. Finally, a myasthenia gravis–type condition after acute infection or vaccination has been reported, which can sometimes cloud the differential diagnosis between infection and new onset or exacerbation of disease [22]. We could hypothesize that this may be due to autoantibodies generated by nAChRs during SARS-CoV-2 virus infection, resulting in autoimmunity to the nAChR after infection.

Our results indicate that varenicline interacts with the RBD of the S glycoprotein of SARS-CoV-2. This is consistent with data from in silico studies that suggest that varenicline binds directly to the hinge side of this region with high affinity [23]. This binding may prevent a change in the S protein to the up-conformation and inhibit subsequent binding by the ACE2 and/or nAChR binding site [23], thus potentially preventing or reducing host cell infection. In studies conducted with the omicron variant (B.A.1) of the S protein, experiments suggest that the S protein probably binds to the α4β2 nAChR and facilitates, by an allosteric mechanism (given no displacement with an increased agonist concentration, and the reduction in amplitude and slope), the activation of the receptors by ACh. These findings have important implications in the pathophysiology of SARS-CoV-2 viral infection because modulation of the α4β2 nAChR throughout the body could have important implications for various functions, including neuronal transmission. Compared with experiments performed with the alpha variant (B.1.17), investigating desensitization of the α4β2 nAChR revealed that the omicron variant S protein caused no difference in the desensitization properties to low concentrations of varenicline, although there was a potentiation of the inward current after preincubation. These findings suggest that the viral mutations in the S protein may have changed the affinity for interacting with the nicotinic agonist varenicline.

Although the studies reported here were exploratory and therefore interpretation is limited, we have confirmed that the nAChR agonist varenicline, the active ingredient of Tyrvaya nasal spray and Chantix/Champix, may have the potential to interact with and inhibit interaction with the alpha variant (B.1.17) S protein, although the binding site seems to have been lost with the mutation to the omicron variant (B.A.1), possibly due to a mutation in the amino acid sequence. Further, the nicotinic cholinergic system has been postulated to be involved in the pathophysiology of severe COVID-19 due to immune dysregulation and cytokine storm because the cholinergic anti-inflammatory pathway may be an important regulator of the inflammatory response [8,9]. Finally, given the in vitro and in vivo effectiveness seen in this and other recent studies [14], we propose that varenicline nasal spray warrants further investigation as an antiviral agent for pre-/postexposure prophylaxis and/or prevention of transmission of SARS-CoV-2.

## 4. Materials and Methods

Oocytes were obtained from mature *Xenopus laevis* females weighing 150 to 300 g and housed in groups of three to five per vivarium at 20–22 °C under exposure to daylight with artificial light supplementation (6 AM–8 PM in summer; 8 AM–7 PM in winter). Ovaries were harvested from deeply anesthetized animals by cooling at 4 °C and tricaine methanesulfonate (MS-222 at a concentration of 150 µg/L) in sodium bicarbonate (300 µg/L) (according to the latest rules adopted by the FSVO). Once anesthetized, each animal was sacrificed (decapitated) in compliance with the Animal Welfare Act and other federal statutes and regulations relating to animals in medical research. A small piece of the ovary was isolated from each sample for immediate preparation and the remaining part was placed at 4 °C in a sterile Barth’s solution containing the following (in mM): NaCl (88), KCl (1), NaHCO_3_ (2.4), HEPES (10), MgSO_4_.7H_2_O (0.82), Ca(NO_3_)_2_.4H_2_O (0.33), and CaCl_2_.6H_2_O (0.41) at pH 7.4, and supplemented with 20 µg/mL of kanamycin and 100 unit/mL penicillin.

### 4.1. Human nAChRs

Injections of complementary DNA encoding for the human α4β2, α3β4, α3α5β4, α4α6β2, and α7 receptors were performed in ≥95 oocytes using an automated injection device according to the manufacturer’s instructions [24]. Receptor expression was examined ≥2 days later.

### 4.2. SARS-CoV-2 S Protein

The SARS-CoV-2 Spike-RBD and the S1 and S2 subunits were obtained from Lucerna-Chem AG (Lucerne, Switzerland; #230-01102). The SARS-CoV-2 Spike-RBD and the S1 and S2 subunits were separately dissolved in a solution of imidazole 150 mM. The SARS-CoV-2 B.1.1.529 (omicron) Spike-RBD was obtained from Lucerna-Chem AG (from Sino Biological #40592-V08H121) in lyophilized form.

### 4.3. Varenicline Tartrate

Varenicline tartrate was obtained from Tocris Bioscience (Geneva, Switzerland).

### 4.4. Electrophysiological Recordings

All recordings were performed at 18 °C and cells perfused with OR2 medium containing the following (in mM): NaCl (88), KCl (2.5), HEPES (5), CaCl_2_.2H_2_O (1.8), and MgCl_2_.6H_2_O (1) at pH 7.4. Oocytes were voltage clamped at –80 mV (unless otherwise indicated) throughout the experiment. Currents were evoked by ACh prepared as a concentrated stock solution (10^–1^ M) in water and then diluted in the recording medium to obtain the desired test concentration. Data were captured and analyzed using a proprietary data acquisition and analysis platform (HiQScreen Sàrl, Geneva, Switzerland) running under MATLAB (MathWorks, Inc., Natick, MA, USA). Control non-injected cells display no detectable response to ACh.

### 4.5. SARS-CoV-2 B.1.1.7 S Protein Interaction with Human nAChR Subtypes

**Figure 11:**
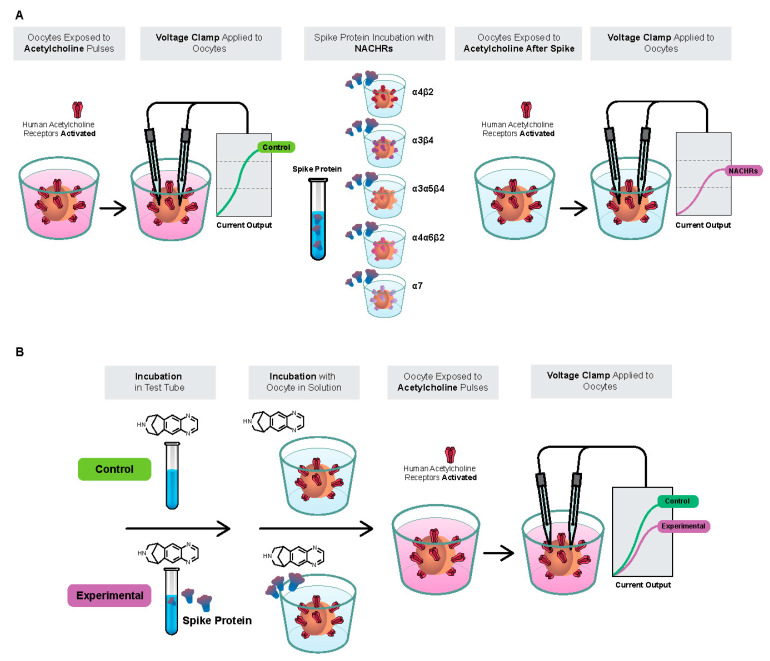
(**A**) Experimental protocol to assay effects of spike protein containing the receptor-binding domain (Spike-RBD) with human nicotinic acetylcholine receptor (nAChR) subtypes. (**B**) Experimental protocol to assay effects of varenicline with Spike-RBD at human α4β2 nAChRs.

A series of experiments were performed to characterize the interaction of the SARS-CoV-2 B.1.1.7 Spike-RBD, S1, and S2 proteins with human α4β2, α4α6β2, α3β4, α3α5β4, and α7 nAChRs. The experimental protocol to assay the putative effects of the Spike-RBD protein at these receptors is shown in Figure 11 (note that the putative effects of the vehicle were assessed before testing the RBD and caused no detectable activity). Briefly, the system loaded the oocyte and assessed the membrane properties and response to a brief ACh test pulse. Cells displaying robust ACh-evoked currents and adequate membrane properties were kept for subsequent measurements. Next, cells were first challenged by brief ACh test pulses applied in growing concentrations. After these recordings, the cell was incubated for 225 s in 1 µg/mL of the Spike-RBD (or S1 or S2) protein before each cycle of ACh pulse testing. Altogether, the cell was maintained in the presence of the Spike-RBD (or S1 or S2) protein for up to 1 h.

### 4.6. SARS-CoV-2 B.1.1.529 (Omicron Variant) S Protein Interaction with Human α4β2 nAChRs

A series of experiments were performed to characterize the interaction of the SARS-CoV-2 B.1.1.529 Spike-RBD proteins with human α4β2 nAChRs. Similar to the protocol above, cells were incubated for 225 s in 1 µg/mL of the SARS-CoV-2 B.1.1.529 Spike-RBD protein before each cycle of ACh pulse testing.

### 4.7. Varenicline Interaction with Spike-RBD and SARS-CoV-2 B.1.1.529 Spike-RBD

Previous studies have shown that varenicline causes desensitization of the α4β2 nAChR [25]. The desensitization profile of the α4β2 nAChR is highly predictable and reproduceable. Based on this interaction, it is possible to use the observed desensitization of α4β2 nAChRs as a functional electrophysiological tool to evaluate how nicotinic agonists interact with Spike-RBD or SARS-CoV-2 B.1.1.529 Spike-RBD. Comparing the effects of incubation with varenicline alone or with varenicline preincubated with the Spike-RBD/SARS-CoV-2 B.1.1.529 Spike-RBD protein allows for the assessment of the potential interaction of varenicline with a fixed concentration of the Spike-RBD protein. Should a significant interaction be observed, this would provide insight into the mechanism of action and rationale for investigating nAChR agonists in preventing or treating SARS-CoV-2 infections.

In this experiment, oocytes were exposed to low, sustained applications of varenicline to demonstrate desensitization of the human α4β2 nAChR to ACh. Currents evoked by brief ACh test pulses (50 mM) recorded in a cell expressing the complementary DNA encoding for the human α4β2 nAChR were recorded first in control conditions and then during exposure to a series of varenicline concentrations ranging from 0.03 to 300 nM. This series of experiments was then repeated in another group of cells, but each varenicline solution was first mixed with 1 µg/mL Spike-RBD or 10-μg/mL SARS-CoV-2 B.1.1.529 Spike-RBD and incubated for ≥1 h before the experiment to allow investigation of the possible interaction between varenicline and Spike-RBD.

## Data Availability

The data sets generated during and/or analyzed during the current study are not publicly available but are available from the corresponding author upon reasonable request.
